# Enhancing the expression of a key mitochondrial enzyme at the inception of ischemia-reperfusion injury can boost recovery and halt the progression of acute kidney injury

**DOI:** 10.3389/fphys.2023.1024238

**Published:** 2023-02-08

**Authors:** Peter R. Corridon

**Affiliations:** ^1^ Department of Immunology and Physiology, College of Medicine and Health Sciences, Khalifa University, Abu Dhabi, United Arab Emirates; ^2^ Healthcare Engineering Innovation Center, Khalifa University, Abu Dhabi, United Arab Emirates; ^3^ Center for Biotechnology, Khalifa University, Abu Dhabi, United Arab Emirates; ^4^ Indiana Center for Biological Microscopy, Indiana University School of Medicine, Indianapolis, IN, United States

**Keywords:** IDH2 [isocitrate dehydrogenase 2 (NADP+) and mitochondrial], gene delivery, acute kidney injury, hydrodynamic injections, ischemia-reperfusion injury (I/R), hydrodynamic gene delivery

## Abstract

Hydrodynamic fluid delivery has shown promise in influencing renal function in disease models. This technique provided pre-conditioned protection in acute injury models by upregulating the mitochondrial adaptation, while hydrodynamic injections of saline alone have improved microvascular perfusion. Accordingly, hydrodynamic mitochondrial gene delivery was applied to investigate the ability to halt progressive or persistent renal function impairment following episodes of ischemia-reperfusion injuries known to induce acute kidney injury (AKI). The rate of transgene expression was approximately 33% and 30% in rats with prerenal AKI that received treatments 1 (T_1hr_) and 24 (T_24hr_) hours after the injury was established, respectively. The resulting mitochondrial adaptation *via* exogenous IDH2 (isocitrate dehydrogenase 2 (NADP+) and mitochondrial) significantly blunted the effects of injury within 24 h of administration: decreased serum creatinine (≈60%, *p* < 0.05 at T_1hr_; ≈50%, *p* < 0.05 at T_24hr_) and blood urea nitrogen (≈50%, *p* < 0.05 at T_1hr_; ≈35%, *p* < 0.05 at T_24hr_) levels, and increased urine output (≈40%, *p* < 0.05 at T_1hr_; ≈26%, *p* < 0.05 at T_24hr_) and mitochondrial membrane potential, Δψ_m_, (≈ by a factor of 13, *p* < 0.001 at T_1hr_; ≈ by a factor of 11, *p* < 0.001 at T_24hr_), despite elevated histology injury score (26%, *p* < 0.05 at T1_hr_; 47%, *p* < 0.05 at T_24hr_). Therefore, this study identifies an approach that can boost recovery and halt the progression of AKI at its inception.

## 1 Introduction

Maintaining kidney health for its multitude of essential regulatory functions remains a significant global clinical challenge (Ferguson et al., 2008; Corridon et al., 2017; Corridon, 2021; Pantic et al., 2022b). As a result, renal dysfunction is both a common and progressive problem, threatening the lives of millions daily. This complex disorder can stem from prerenal, renal, and postrenal conditions and generally results from renal trauma, blood loss, and the accumulation of various toxins, such as broad-spectrum antibiotics, chemotherapeutic drugs, and radiocontrast agents (Nagase et al., 1977; Thadhani et al., 1996; Bellomo et al., 2004; Cerda et al., 2008; Himmelfarb et al., 2008; Hingorani et al., 2009; Lattanzio and Kopyt, 2009; Kinsey and Okusa, 2011a). Such dysfunction can be gradual or sudden, and it is imperative to address these issues and minimize secondary organ damage, which can occur primarily as a consequence of an acute disorder.

Acute kidney injury (AKI) management depends on identifying and treating its underlying cause(s) (Palevsky et al., 2008; Basu et al., 2012). Fortunately, in some instances, sudden impairment, which is the focus of this work, can be reversed within several weeks to months if the underlying cause has been treated. Yet, beyond certain thresholds, these disorders induce persistent cell death and progressing degrees of atrophy that drive the progression of AKI (Holderied et al., 2020). At this stage, current treatment regimens, which comprise fluid, electrolyte, and acid-base balance management, are still mainly supportive and unable to halt the progression of the illness (Kinsey and Okusa, 2011b; Rostami et al., 2022). As a result, gene therapy has been proposed as an alternative to treat and prevent the underlying causes and progression of AKI (Humes et al., 1997; Torras et al., 2008; Molitoris et al., 2009).

The first human gene therapy trial in 1990 laid the foundation for a new medical paradigm (Imai, 2003). However, since then, gene therapy has undergone more than thirty years of trials and tribulations with unexpected side effects dampening the enthusiasm for this form of treatment (Imai, 2003; Davis and Park, 2019). Fortunately, in the past few years, however, gene therapy has enjoyed a renaissance with recent successes in lymphoma treatment (Nogrady, 2018), retinal degeneration (Leroy et al., 2022), and neuromuscular atrophy (Ali et al., 2021), using a variety of viral and non-viral vector-based applications. With these successes, continued efforts are being employed using a broad spectrum of vectors for proof-of-concept and therapeutic approaches in nearly every organ system in the body, including the kidney. However, the progress in the development of direct gene transfer methods in the kidney has been limited compared with other organs due in part to our lack of knowledge about the biological factors needed to promote efficient vector entry into the cell and the complex anatomical structure of the kidney (Davis and Park, 2019).

Numerous methods have been proposed to deliver exogenous genes to mammalian cells to study and treat human disease (Friedmann and Roblin, 1972; Amiel et al., 2000; Niidome and Huang, 2002; Chen et al., 2003; Appledorn et al., 2008; Miyazaki et al., 2009; Corridon et al., 2013; Collins and Thrasher, 2015; Hitchman et al., 2017; Kolb et al., 2018; Shen et al., 2018; Peek and Wilson, 2022). With specific regard to the kidney, attempts have been made to protect and repair renal function by targeting single genetic loci (Lien and Lai, 1997). Such approaches have utilized purified protein products, plasmids, and viruses encoding peptides and proteins. Historically, recombinant growth factors have been used in experimental and clinical AKI settings to preserve renal function and accelerate tissue repair. For instance, these studies have suggested that hepatocyte growth factor (HGF) may have a significant role in the management of AKI. HGF has been shown to have diverse functions in kidney repair following acute injury, as it can act as both a renotropic and anti-fibrotic agent (Dai et al., 2002; Yazawa et al., 2004). Parallel studies have shown that HGF may prevent cyclosporin-induced tubulointerstitial fibrosis, indicating its additional renoprotective capacity (Yazawa et al., 2004).

Similarly, other investigations have shown that exogenous vascular endothelial growth factor (VEGF) enhances renal microvascular integrity and function following acute and chronic injuries (Corridon et al., 2006; Leonard et al., 2008; Engel et al., 2019; Gao et al., 2020). The therapeutic potential of recombinant interleukin binding protein (IL-18 Bp) was also investigated in established ischemia AKI rodent models. Intravenous injections of IL-18 Bp improved renal function and tubule morphology and reduced tubular necrosis and apoptosis (Wang et al., 2012). More recently, inhibition of IL-18 by peritoneal doses of IL-18 Bp has been shown to reduce renal fibrosis following IRI (Liang et al., 2018). Recombinant uteroglobin treatment prevented glomerulonephritis by reducing proteinuria and cellular pathogenic globulin-glomerular biding (Lee et al., 2004). However, several factors may limit the clinical benefit of this therapy. Such factors include the relatively short half-life of recombinant proteins and the prohibitive costs and recurrent doses required for treatments (Mizui et al., 2004; Yazawa et al., 2004). Altogether, these factors outline a basis for generating adverse side effects that can result from administering supraphysiologic doses of recombinant proteins.

In contrast, vector-based gene transfer procedures can be more straightforward, safer, and cheaper, requiring less frequent dosing (Mizui et al., 2004). For instance, researchers have utilized adenoviral, adeno-associated viral, and lentiviral vectors for gene transfer. Previous studies have identified the ability to robustly elicit targeted gene transfer with adenoviral vectors using pressurized renal vein injection techniuqes (Corridon et al., 2013; Corridon et al., 2021). This type of research has also identified the possibility of improving renal transplant outcomes in clinically relevant models of acute rejection using immunomodulating genes like interleukin-13 (a known potent anti-inflammatory agent) (Sandovici et al., 2007) and 2,3-indoleamine dioxygenase (a stimulator of regulatory T cell production) (Vavrincova-Yaghi et al., 2011) using an adenovirus-based approach *via* intrarenal and renal arterial injections, respectively. These findings are significant since ischemic and toxic renal injuries repair critically depends on regulating a redundant, interactive network of cytokine and growth factors (Wang et al., 2012).

Recent studies have highlighted the benefits and drawbacks of adeno-associated viruses and lentiviruses (Rubin et al., 2019). For example, retrograde ureteral and subcapsular adeno-associated injections have been used to transduce the kidney. However, viral particles leaked from the organ during these processes and mediated substantial off-target gene transfer. Analogously, similar results were observed with lentiviral vectors, which also effectively transduced kidney cells, albeit with less off-target tissue transduction. Thus, it would be of value to devise a system that can modulate gene expression levels in a therapeutic manner capable of reinstating renal function without inducing harmful viral-derived toxicity. However, viral vectors may ultimately be confined to experimental gene therapy applications unless we overcome the obstacles that limit their widespread use (DeYoung and Dichek, 1998; Kay et al., 2001).

Comparatively, one of the simplest forms of DNA transfection involves transfer without a specific vector or carrier molecule. Studies dating back roughly two decades have shown how plasmid DNA can be transferred using only a solvent intravenously (Liu et al., 1999). Plasmid-based vector treatments have confirmed the renotherapeutic potential of HGF as it mediated tissue regeneration and protected tubular epithelial cells from injury and apoptosis during acute renal failure. These results were obtained using single intravenous injections of plasmids encoding HGF (Dai et al., 2002). One major issue related to this intravenous approach was the offsite nature of delivery to the liver. To enhance the delivery to other tissues, namely the kidney, a form of pressurized injection (hydrodynamic-based delivery) was coupled with DNA polyplexation techniques to improve the effectiveness of the technique (Tros de Ilarduya et al., 2010). Even though these tools increased transfection efficiency compared to naked DNA transfections (Hayat et al., 2019), this process could not limit offsite genetic alterations (Rubin and Barry, 2020). Nevertheless, the original hydrodynamic injections technique showed great promise in advancing an ability to aid effective renal gene transfer. Altogether, these studies highlight the generational issues related to combining the appropriate vector with the delivery technique to provide efficient and targeted renal gene therapy.

In particular, hydrodynamic-based delivery increases the permeability of endothelial (Suda and Liu, 2007) and epithelial (Wang et al., 2000) junctions by non-adversely enhancing poration in plasma membranes. This transient process facilitates the cellular internalization of macromolecules of interest (Herweijer and Wolff, 2007). It has also been shown that the unique renal anatomy provides various innate delivery paths (renal artery, renal vein, and ureter) that may be ideal for hydrodynamic gene delivery (Xing et al., 2009), recalling the ability to facilitate efficient exogenous protein expression *in vivo* using plasmid reporter vectors (Corridon et al., 2013; Corridon et al., 2021). Based on these tenets, our group devised ways to examine the ability to alter functional protein activity with plasmid-based vectors by adapting the injection process to include a brief period of vascular cross-clamping. This adaptation focused on limiting or eliminating offsite genetic alterations, resulting in efficacious and targeted exogenous protein expression (Corridon et al., 2013).

After that, we aimed to alter functional protein activity that can be clinically significant to AKI. Specifically, this approach was formulated to induce benefits from ischemic pre-conditioning, which renders tissues resistant to subsequent deleterious effects of prolonged ischemia after previous exposure to vascular occlusion (Ishida et al., 1997). This pre-conditioning effect is linked to an innate organ-derived ability to withstand additional ischemic events after a non-lethal event (Murry et al., 1986). We have previously shown, using proteomic screens, that this non-lethal approach facilitates the upregulation of key mitochondrial proteins, including NADP + -dependent isocitrate dehydrogenase 2 (IDH2) (Kolb et al., 2018). IDH2 is the primary enzyme responsible for generating the mitochondrial NADPH pool critical for maintaining the mitochondrial antioxidant system (Lee et al., 2017). This system helps protect cells from potentially harmful reactive oxygen species. These results, in turn, provided a potential genetic target of clinical significance that may be used to alter proteomic expression using hydrodynamic-based approaches for therapeutic purposes.

Moreover, prior research has shown that rodents deficient in IDH2 observed a unique acceleration of renal senescence and substantial deterioration in structure and function *via* disruptions to redox status that promoted oxidative damage and apoptosis (Lee et al., 2017). Similar reductions in redox status are well-established consequences of ischemia-reperfusion injury (IRI), leading to AKI (Zhang et al., 2021). Yet, from a therapeutic point of view, we have previously altered the mitochondrial proteome *via* hydrodynamic IDH2 plasmid delivery. Such alterations have significantly upregulated IDH2 renal expression and conferred organ-wide protection against subsequent IRI in live rats in a manner that mimics IPC. Such focus on elucidating IDH2’s protective role in that study also helped uncover how hydrodynamic IDH2-mediated exogenous expression increased the mitochondria membrane potential (Δψ_m_), maximal respiratory capacity, and intracellular ATP levels. Based on the success of these results, it is of value to extend the utility of hydrodynamic renal gene therapy by determining whether upregulating the expression of this key mitochondrial enzyme could boost recovery and halt the progression of AKI at the inception of such injury. Thus, herein, prerenal AKI was induced in rats using an IRI model, which is widely applied for both fundamental and therapeutic intervention studies of AKI (Le Clef et al., 2016), to help examine the associated therapeutic potential of hydrodynamic-based delivery known to elicit efficacious and targeted genetic alterations.

## 2 Materials and methods

### 2.1 Prerenal acute kidney injury

**FIGURE 1:**
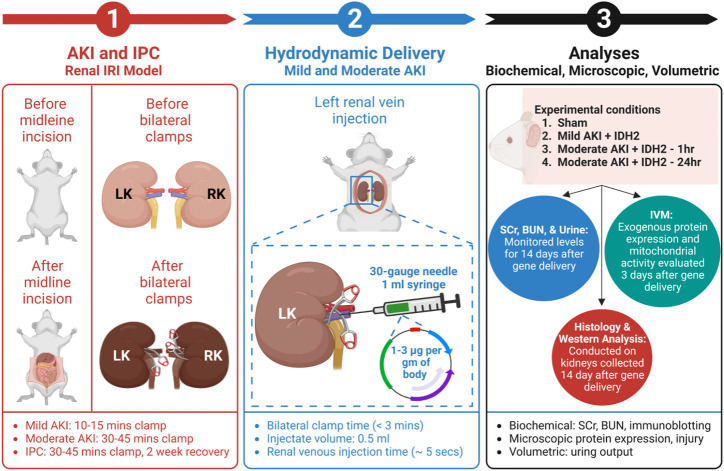
An outline of the experimental workflow. (1) This experimental approach began by subjecting animals to different degrees of IRI to induce AKI *via* bilateral renal pedicle clamps for periods to induce mild (occluded blood flow for 10–15 min) and moderate (occluded blood flow for 30–45 min) injuries, and IPC (occluded blood flow for 30–45 min). (Corridon et al., 2013) (2) The next step included hydrodynamic gene delivery for four experimental conditions by conducting rapid retrograde injections using vascular cross-clamping into the renal vein. The experimental conditions were sham injury, mild injury followed by hydrodynamic gene delivery 1 h after reperfusion (Mild AKI + IDH2—1h), moderate injury followed by hydrodynamic gene delivery 1 h after reperfusion (Moderate AKI + IDH2—1h), and moderate injury followed by hydrodynamic gene delivery 24 h after reperfusion (Moderate AKI + IDH2—24 h). The injections took approximately 5 s to deliver 1–3 µg per Gram of plasmid DNA per body weight suspended in 0.5 mL saline (Engel et al., 2019). (3) The last phase experiment relied on a series of biochemical, microscopic, and volumetric analyses to examine whether hydrodynamic gene delivery could upregulate IDH2 expression in the mammalian kidney and what effects altering the mitochondrial proteome in this manner could have on serum creatinine (SCr), blood urea nitrogen (BUN), and urine output levels, as well as the renal mitochondrial membrane potential (Δψ_m_) and renal microarchitecture. LK, left kidney; RK, right kidney; IVM, intravital microscopy; and IDH2, isocitrate dehydrogenase [NADP], mitochondrial.

Male Sprague Dawley rats, 200–250 gm, were obtained from Harlan Laboratories (Indianapolis, IN, United States) and allowed free access to food and water. After anesthetization by intraperitoneal injections of sodium pentobarbital (50 mg/kg body weight), each animal was placed on heating pads to maintain normal physiological temperature. Midline incisions were performed to expose the renal hila. In this study, rats were subjected to a sham injury or either of two forms of AKI: mild and moderate. First, to induce a mild injury, the micro-serrefine clamps with delicate, atraumatic serrations (Fine Science Tools, Foster City, CA, United States) were used to bilaterally clamp the renal pedicles for 10–15 min to induce a mild injury. Second, the clamping period was 30–45 min to generate a moderate injury. The clamps were removed at the end of each injury induction period to reinstate renal blood flow and generate IRI. Last, animals in the sham (control) group underwent the same procedures without having their renal pedicle clamped. It should be noted that during the actual or sham clamping periods, the midline incisions were temporarily closed to prevent infection and support homeostasis. A schematic of the experimental workflow is presented in Figure 1.

### 2.2 Ischemic pre-conditioning

Similar to the induction of AKI, rats were again anesthetized in the manner described above and subjected to bilateral pedicle clamps to occlude renal blood flow for 30–45 min for IPC. The incisions were again temporarily closed during ischemia. The animals were then allowed to recover for 14 days, after which *in vivo* mitochondrial activity was examined. For such studies, kidneys were collected and analyzed for adaptations in mitochondrial activity and compared to control rats using immunoblotting techniques.

### 2.3 Serum creatinine and blood urea nitrogen measurements

Blood samples were collected from the rats in 1 mL Eppendorf heparin-treated tubes after making small incisions on their tails. These samples were centrifuged at 100,000–130,000 rpm for 10 min. The supernatants were then stored at 4°C. Quantitative determination of creatine kinase activity in serum was then estimated with Pointe Scientific CK (Liquid) Reagents (Point Scientific, Inc., Canton, MI, USA). Serum creatinine (SCr) measurements were performed with a Beckman Creatinine Analyzer 2 (Beckman Instruments, Brea, CA, USA) according to the manufacturer’s specifications and reported in milligrams per deciliter (mg/dL). Sera blood urea nitrogen (BUN) measurements were made using the Liquid Urea Nitrogen Reagent Set (Pointe Scientific, Canton, MI, United States), whereby the working reagent was prepared by mixing five parts of the enzyme reagent (R1) with 1 part of coenzyme (R2) reagent. Approximately 10 μL of each serum sample was added to the working reagent (1,000 μL), and the absorbance was immediately measured using a microplate reader. The levels of these metabolites were measured before all forms of IRI were induced and monitored across the subsequent 14 days to investigate the effect of IDH2 hydrodynamic-based injections.

### 2.4 Urine output measurements

Rats were also housed separately in metabolic cages (Fisher Scientific, Hampton, NH, USA) to allow standard volumetric measurements of individual urine outputs collected daily in falcon tubes without intervention. The animal committee approved the studies using the metabolic cages, and the animals were checked daily and independently by the researcher and the animal housing staff for signs of stress; if any adverse conditions were identified, they would have been excluded from the study.

### 2.5 Intravital multiphoton fluorescence microscopy

Intravital microscopy (IVM) and its fundamentals are well outlined in the literature (Burne-Taney et al., 2005; Molitoris and Sandoval, 2005; Tanner et al., 2005; Ashworth et al., 2007; Dunn et al., 2007; Corridon et al., 2013; Hall et al., 2013; Corridon et al., 2021; Cai et al., 2022; Corridon, 2022a; Shaya et al., 2022). However, briefly, after sedation, vertical flank incisions externalized each rat’s left kidney, which was then placed inside a glass bottom dish containing saline. This imaging dish was set above a X60 water immersion objective which supported the acquisition of fluorescent micrographs from an inverted Olympus FV 1000-MPE microscope equipped with a Spectra-Physics MaiTai Deep See laser tuned to an 800–850 nm excitation wavelength (Olympus, Tokyo, Japan). The system was connected to external detectors for multiphoton imaging, and dichroic mirrors for the collection of blue, green, and red emissions.

### 2.6 Hydrodynamic injections

In this study, the following four experimental groups of rats were subjected to hydrodynamic injections performed with vascular cross-clamping: sham injury; mild injury followed by hydrodynamic gene delivery 1 h after reperfusion; moderate injury followed by hydrodynamic gene delivery 1 h after reperfusion; and moderate injury followed by hydrodynamic gene delivery 24 h after reperfusion. For this injection process, midline incisions allowed the isolation of the left renal vein, with a 4–0 silk loop, in each sedated rat. The renal artery was occluded first and followed by the renal vein, using the micro-serrefine clamps. The vein was then gently elevated, and 0.5 mL infusates were rapidly injected distal to the clamp using a 30-gauge needle within roughly 5 s. Pressure was then applied to the injection site for approximately 3 min to induce hemostasis. The venous clamp was removed, followed by the arterial clamp, and the animal was prepared for recovery.

The various infusates were prepared as follows. For the toluidine blue infusates, stock solutions were prepared by dissolving 50 mg of tolonium chloride dye (Toluidine Blue O, Electron Microscopy Sciences, Fort Washington, PA, United States) in 5 mL of 0.9% saline, of which 0.5 mL was used for each injection. This infusate was used to examine whether this targeted injection process could support exogenous macromolecular confinement to the injected kidney.

Infusates of fluorescent dextrans comprised of 0.5 mL saline containing 4 kDa (low molecular weight) fluorescein isothiocyanate, FITC, and 150 kDa (large molecular weight) Tetramethylrhodamine-isothiocyanate, TRITC, dextrans (TdB Consultancy, Uppsala, Sweden), and 30 μL of Hoechst 33,342 (Invitrogen, Carlsbad, CA, United States), which was applied to identify cellular nuclei within the tubular epithelium). These infusates were used to investigate the subcellular incorporation of the exogenous macromolecules into the renal epithelium, as well as renal structure and function in the injury models.

Fluorescent plasmid DNA infusates were used to investigate live and subcellular incorporation of the exogenous macromolecular and renal structure and function in the injury models. These fluorescent infusates were prepared by suspensions in saline using Qiagen Maxi Prep systems (Qiagen, Chatsworth, CA, United States). These plasmids encoded enhanced green fluorescent protein (EGFP)-actin (Clontech Laboratories, Inc., Mountain View, CA, United States). This pAcGFP1-actin vector expressed the Aequorea coerulescens GFP(AcGFP1)-actin fusion protein in mammalian cells. The protein is incorporated into growing actin filaments and allows for the visualization of actin-containing subcellular structures. Likewise, non-fluorescently labeled plasmid vectors that encode mitochondrial enzymes isocitrate dehydrogenase [NADP], mitochondrial, IDH2, (OriGene Technologies, Inc., Rockville, MD, United States) were also used in this study. The IDH2 vector was a Myc-DDK-tagged ORF clone of *Homo sapiens* isocitrate dehydrogenase 2 (NADP+), mitochondrial (IDH2), nuclear gene encoding mitochondrial protein as transfection-ready DNA. Moreover, each transgene solution was prepared by suspending 1–3 µg of plasmid DNA per Gram of body weight of either plasmid vectors in 0.5 mL of saline, as outlined in previous related studies (Corridon et al., 2013; Collett et al., 2017; Kolb et al., 2018; Corridon et al., 2021).

### 2.7 *In vivo* estimations of exogenous gene expression

In order to estimate the degree of transgene expression that resulted from the pressurized injection process, intravital micrographs were used to determine the proportion of renal segments that expressed the transgenes. For this process, micrographs were acquired from three adjacent intravital fields and were randomly chosen from each live kidney within a given experimental group. A segment was considered transfected if at least one of its cells expressed the EGFP-actin plasmid vector. Using ImageJ software (National Institute of Mental Health, NIH, Bethesda, MD, United States), transgene fluorescence signals were identified with intensities at least double those of autofluorescent signals. With this criterion, the percentage of segments that expressed the reporter transgenes within fields acquired with the X60 objective was calculated as the average percentage of transfected segments within the randomly chosen nephron cross-sections.

### 2.8 *In Vivo* estimations of the renal mitochondrial membrane potential

To study *in vivo* mitochondrial activity, a stock solution of tetramethyl rhodamine methyl ester, TMRM (Invitrogen Molecular probes, Eugene, OR, United States) was prepared by suspending 5 μg of the dye in 2 mL of saline. An incision was made to expose the jugular vein. The vein was isolated with two 3–0 or 4–0 silk loops. The superior loop was tied and clamped with a pair of hemostats to stiffen and elevate this vein. A PE-50 polyethylene catheter tubing (Clay Adams, Division of Becton, Dickson and Company, Parsippany, NJ, USA) was attached to a 1 mL syringe containing the TMRM injectate was inserted into a minor incision in the jugular vein. The other silk loop was used to anchor the catheter in the vein. The plasma membrane-permeant TMRM dye was slowly infused into the vein, and intravital imaging was performed once a steady state was established to estimate the renal mitochondrial membrane potential (Δψ_m_). To estimate renal mitochondrial membrane potential (Δψ_m_), mean fluorescent intensities from TMRM-labelled mitochondria were computed from each tubule within three adjacent intravital fields randomly chosen from each live kidney within a given experimental group using ImageJ software.

### 2.9 Histological assessments

After fixation with 4% paraformaldehyde for 24 h at 4°C, the kidney samples were immersed in formalin, which was phosphate-buffered, for 24 h. The samples were kept at room temperature and then rinsed with distilled H_2_O. After washing, they were stored in 70% ethanol at room temperature and then subjected to dehydration *via* a gradation of ethanol solutions that ranged from 70%–100%, with increments of 10%. Clearing with xylene and infiltration with paraffin (four paraffin changes under vacuum at 59°C for 45 min each) followed. The samples were then embedded in fresh paraffin for sectioning to produce around 5 μm thick sections with a Reichert-Jung 820 microtome (Depew, NY, United States). These sections were counterstained with hematoxylin and eosin (H&E) and imaged with an X60 objective mounted on a Nikon Microphot SA Upright Microscope (Nikon, Tokyo, Japan). The degree of injury associated with each treatment condition was estimated as a percentage of the injured cells within the micrographs collected. Specifically, these percentages were computed as ratios of cells with disrupted/adversely altered plasma membranes to the total number of cells in a micrograph and averaged across three randomly chosen micrographs for each animal.

### 2.10 Western blot analysis

Whole kidneys were collected from anesthetized rats and cryofixed in liquid nitrogen. Post cryofixation, transverse sections of roughly 300 mg, extending from the cortex to the pedicle, were obtained and homogenized in 300 µL RIPA buffer using Dounce homogenizer on ice and the lysate was spun at 13,000 g for 20 min in a 4°C pre-cooled centrifuge. Bicinchoninic acid assays were then performed to determine the protein concentration, and denatured protein samples were acquired by adding equal volumes of 2X Laemmli Sample Buffer at 70°C for 5 min. After which 5 ug of each protein sample was loaded on 10% SDS-PAGE gel, along with molecular weight markers, and ran for 50 min at 240 V. Protein samples were then transferred from the gel to the membrane for approximately 30 min at 24 V, which preceded overnight membrane blocking that occurred the at 4°C using TBS blocking solution with 3% FBS.

The next day, the membrane was incubated with 1:1,000 dilution of primary antibody (Rabbit PolyAb Anti-IDH2 (Novus Biologicals, Littleton, CO, United States) in TBS blocking solution with 0.3% FBS for 1 h at room temperature and washed then three times with TBS for 5 min. The membrane was then incubated with 1:40,000 dilution of the secondary antibody HRP D&R in TBS blocking buffer with 0.3% FBS at room temperature for 1 h, and the gel was washed three times with TBS. Finally, the blots were incubated in the enhanced chemiluminescence (ECL) reagents, SuperSignalTM West Pico Chemiluminescent Substrate (Thermo Fisher Scientific, Waltham, MA, United States), to facilitate image acquisition.

### 2.11 Statistical analysis of data

The Kruskal–Wallis one-way analysis of variance (ANOVA) with the *post hoc* pairwise Dunn’s test was applied as appropriate to examine levels of exogenous gene expression, SCr, BUN, urine output, Δψ_m_, and cellular injury. These analyses were conducted at a *p* < 0.05 level of significance and the data are presented as the mean ± SD.

## 3 Results

### 3.1 Hydrodynamic renal fine needle injections support widespread and targeted delivery of exogenous probes in rats with mild and moderate AKI

Whole kidneys were extracted from euthanized rats after they received hydrodynamic delivery of 0.5 mL of toluidine blue dye. These kidneys were harvested and sectioned within 20 min of injections, revealing substantial and localized dye uptake in all four examined conditions:

• sham injury;
• mild injury followed by delivery 1 h after reperfusion;
• moderate injury followed by delivery 1 h after reperfusion; and
• moderate injury followed by delivery 24 h after reperfusion.

**FIGURE 2:**
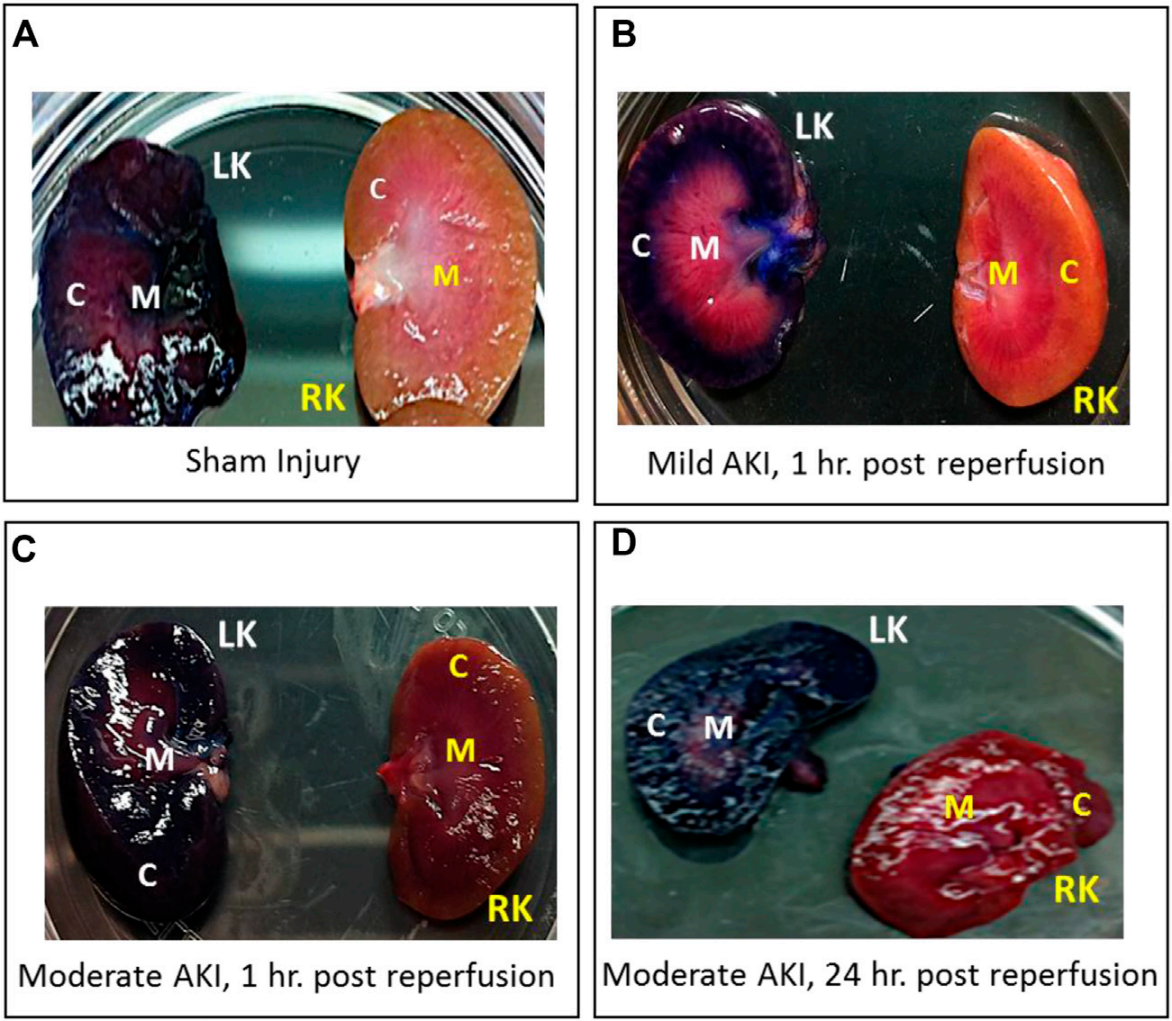
Digital photographs were used to illustrate the localized and widespread delivery resulting from renal vein hydrodynamic injections performed in **(A)** Sham injured rats and those with **(B)** mild AKI (injections performed 1 h after inducing AKI), **(C)** moderate AKI (injections performed 1 h after inducing AKI), and **(D)** moderate AKI (injections performed 24 h after inducing AKI). These kidneys were harvested and sectioned within 20 min of performing the injections. The dye appeared throughout the cortex and medulla of the left kidneys and was absent in the contralateral kidneys. LK = left kidney, RK = right kidney, C = cortex, and M = medulla.

In each case, the dye appeared throughout the cortex and medulla of the left kidneys and was absent in the contralateral kidneys, as shown in various digital photographs (Figure 2). These results illustrate the ability to facilitate localized delivery to the kidney using hydrodynamic-based delivery with vascular cross-clamping in rats with sham and mild injuries and in animals with substantially greater degrees of injury. 

### 3.2 Retrograde hydrodynamic injections facilitate the delivery of large and low molecular weight dextrans in rats with mild and moderate AKI

**FIGURE 3:**
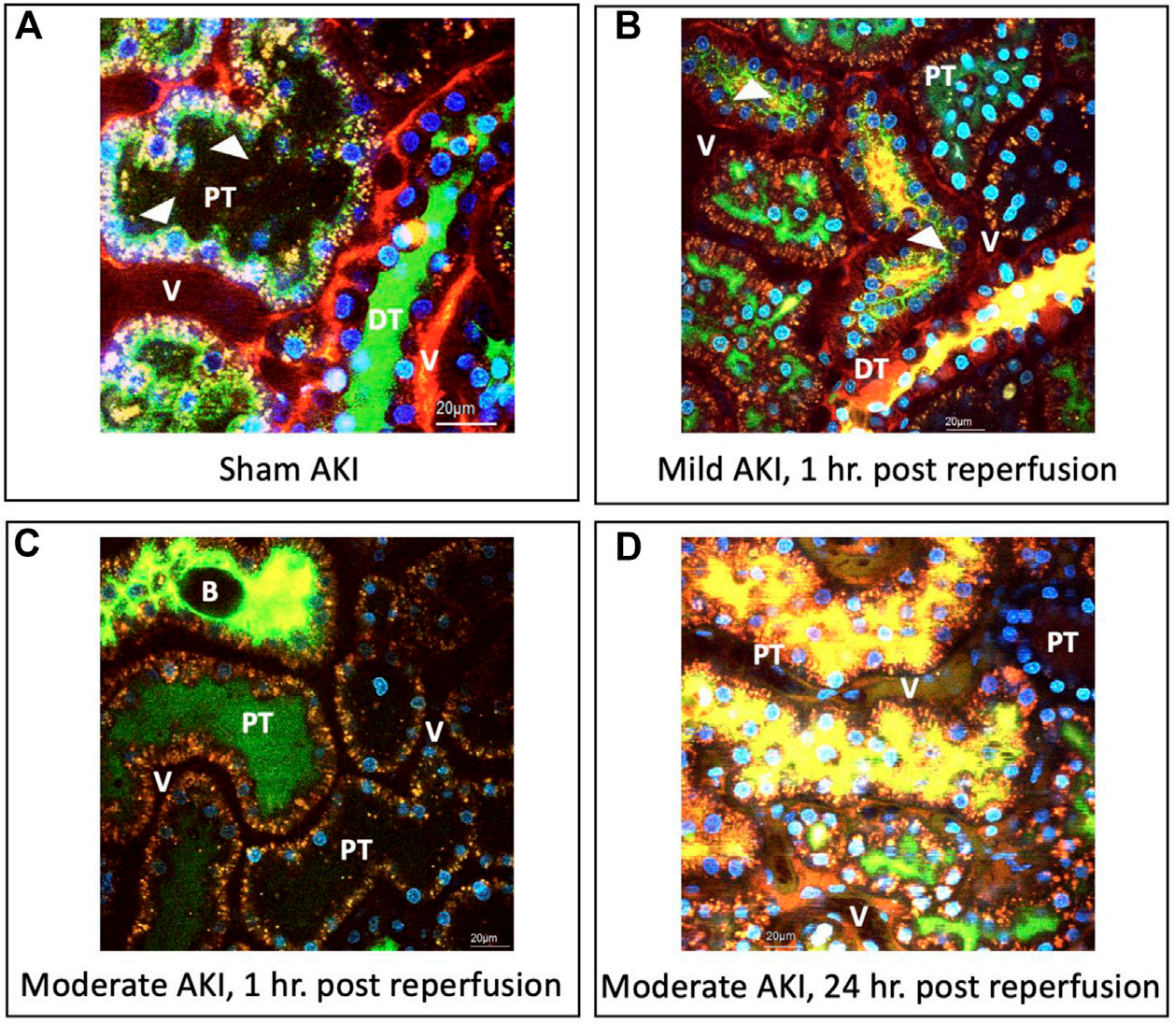
Intravital micrographs illustrate the incorporation of macromolecules by various renal compartments resulting from renal vein hydrodynamic injections performed in **(A)** sham injured rats and those with **(B)** mild AKI (injections performed 1 h after inducing AKI), **(C)** moderate AKI (injections performed 1 h after inducing AKI), and **(D)** moderate AKI (injections performed 24 h after inducing AKI). These micrographs were acquired from live rats within 20 min of receiving hydrodynamic infusions of 0.5 mL saline containing 4 kDa FITC and 150 kDa TRITC dextrans, and Hoechst 33,342 (this image A was acquired with 1.5X digital zoom to illustrate better the dynamic events outlined). The arrowheads highlight regions where the fluorescent dextran molecules appear to bound brush borders or endocytosed puncta within tubular epithelial cells. All images were taken using a blend of the red-, green-, and blue-pseudo-color channels. DT, distal tubule; PT, proximal tubule; and V, vasculature; and B, bleb, observed within the tubular lumen.

Compared to the macroscopic (visible) evidence provided in the previous section, intravital micrographs obtained from sham injured rats (Figure 3A) approximately 20 min after hydrodynamic infusions of infusates with dextrans and Hoechst stains also illustrated the robust uptake of each fluorescent tag. These images were obtained after this period to allow for homeostasis after the injection process. Intense TRITC-derived signals (red-based fluorescence) from the large molecular weight dextran were confined to the vasculature, which is consistent with normal/minimally altered function (Dunn et al., 2003; Molitoris and Sandoval, 2005; Dunn et al., 2007). At the same time, FITC-conjugated low molecular weight dextran molecules lined brush borders. These green, fluorescent molecules appeared as internalized puncta within the proximal tubule epithelium and concentrated within the distal tubular lumen (seen as a substantial degree of the green fluorescence that filled the lumen of this nephron segment). These observations provide evidence of widespread delivery of exogenous materials in the sham group and intact structural and functional renal capacities.

Similarly, there was clear evidence of widespread renal delivery of the exogenous probes observed in animals with mild and moderate forms of injury. For instance, in the mildly injured rats, there was evidence of impaired filtrative capacities based on the simultaneous presence of both FITC and TRITC dyes in the lumens of proximal and distal tubules (Figure 3B), as well as the reduced intensity of the large molecular weight TRITC dextran within the peritubular vasculature. Additionally, the reduced levels of the low molecular weight FITC dextran molecules that normally line the proximal tubule’s brush border highlighted alterations to this epithelium’s innate endocytic capacities. There was also evidence of cellular injury outlined by the low level of Hoechst-labelled nuclei within tubular lumens.

As expected, the injury levels escalated with moderate IRI-derived injuries. There was still a substantial and widespread presence of the FITC and TRITC dyes, yet their localization differed from the sham and mild conditions. In particular, there was a far greater degree of inhomogeneous and composite TRITC and FITC fluorescent-based signals emanating from the peritubular vasculature and lumens illustrated by the reduced levels of fluorescence in the vasculature seen in the fields obtained from the moderately injured rats, 1 h post-reperfusion, and the presence of intense of yellow-based fluorescence, blebs, and cellular nuclei within the lumens (Figure 3C). Likewise, atypical, brown-based fluorescence lined the peritubular vasculature of the moderately injured rats that received hydrodynamic-based injections 24 h post-reperfusion (Figure 3D). The tubular lumens also contained higher levels of TRITC and FITC dye composites and detached nuclei. Such deformation of tubular epithelial nuclei and their atypical presence in the lumen are hallmarks of IRI-induced AKI (Corridon et al., 2021). Again, additional signs of impaired filtration are presented and depicted by the reduced concentrations of FITC molecules and blebs within distal tubule lumens known to stem from IRI (Hall and Molitoris, 2014). Beyond the aspects mentioned above related to injury, it was essential to observe signs of the incorporation of the TRITC dextran molecules in the tubular epithelia. As a result, these images provided evidence that this injection technique can effectively deliver various macromolecules across the nephron in the various injury models.

### 3.3 Intravital microscopy and Western blot analysis demonstrate that hydrodynamic gene delivery induces exogenous renal protein expression and upregulates IDH2 activity in rats with mild and moderate AKI

**FIGURE 4:**
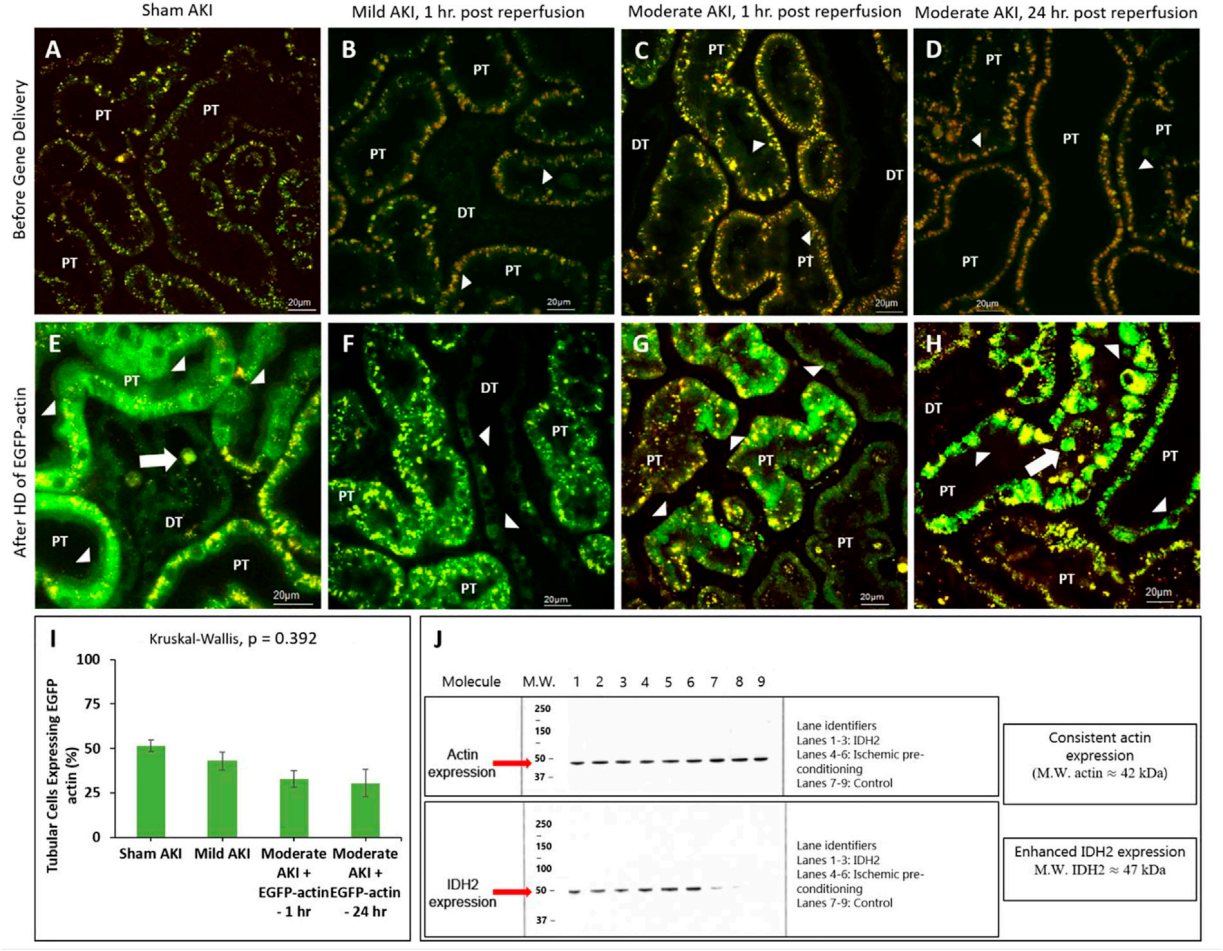
Hydrodynamic injection derived fluorescent actin expression. Intravital micrographs illustrate the hydrodynamic delivery-based expression of EGFP-actin in various settings. In sham rats **(A)** before and **(E)** after gene delivery. In rats subjected to mild AKI (injections performed 1 h after inducing AKI), **(B)** before and **(F)** after gene delivery. In rats subjected to moderate AKI (injections performed 1 h after inducing AKI), **(C)** before and **(G)** after gene delivery. In rats subjected to moderate AKI (injections performed 24 h after inducing AKI), **(D)** before and **(H)** after gene delivery. The arrowheads highlight regions where the fluorescent actin expression is highlighted along the brush border and within cuboidal distal tubule cells. The arrows pinpoint fluorescent casts/debris within the tubular lumen. Image E was acquired with 1.5X digital zoom to highlight these details further. All micrographs were taken 3 days after gene delivery using a blend of the red- and green-pseudo-color channels to distinguish the true presence of EGFP-based fluorescence. **(I)** Comparison of the levels of exogenous gene expression induced by the hydrodynamic technique determined from IVM data. **(J)** Western blots illustrate the enhanced expression of IDH2 in kidney tissues harvested from rats 3 days after hydrodynamic injections of the IDH2 plasmids (lanes 1 through 3) and IPC (lanes 4 through 6) compared to the control group that received sham injections (lanes 7 through 9). *n* = 3 for all groups. DT, distal tubule and PT, proximal tubule.

It should also be noted that the intravital investigations were limited to proximal and distal tubular compartments, as these segments are easily accessible within the IVM system’s 100–200 µm imaging depth, while the superficial glomeruli are rare in the strain of rat. Multiphoton micrographs presented in Figure 4 reveal enhanced EGFP-actin-derived fluorescence signals within proximal and distal tubule epithelia from the sham and AKI model rats 3 days after gene delivery. This time point was chosen based on previous characterizations that outline the potential to induce stable exogenous gene expression as early as this period after gene transfer (Corridon et al., 2013). At that time, distinctive fluorescent patterns were observed along proximal tubule brush borders and within distal tubular epithelial cells of sham (Figure 3E) and mildly injured (Figure 3F) animals. In comparison, these patterns were absent in the renal compartments of rats subjected to moderate IRI. As anticipated, moderate AKI induced substantial disruptions to the typical renal architecture, which provides clear means to differentiate proximal ad distal tubular segments in the strain of rats used for this study. Nevertheless, IRI-derived injury made it challenging to make routine morphological distinctions between proximal and distal segments.

Additionally, the different rates of transgene expression in live renal segments were determined from the percentage of renal segments within a microscopic field that expressed the transgenes (Figure 4I). Again, a segment was considered transfected if at least one of its cells expressed the EGFP fluorescent transgenes. These estimations provided approximately 30%–40% transfection efficiency rates in the superficial cortex accessible by intravital multiphoton microscopy in rats that received moderate IRI (32.8% in the rats that received hydrodynamic gene delivery 1 h after reperfusion and 30.4% in the rats that received hydrodynamic gene delivery 24 h after reperfusion). These estimated efficiencies were lower than those obtained for sham (51.4%) and mildly injured (43.0%) rats, whose transgene expression rates were roughly 50%. However, no significant difference (*p* = 0.392) was detected in the levels of transgene expression among the groups, indicating the potential of this technique to induce genetic alterations throughout the diseased kidney.

Western blot analyses were performed to examine further genetic alterations that could have resulted from hydrodynamic-gene delivery and IPC. These blots are presented in Figure 4J. Control data obtained from the Western analyses illustrate the consistent and intense levels of actin expression in regions that correspond to the molecular weight of actin (≈42 kDa) molecules in all groups. These results are consistent with the innate presence of actin within the kidney. In comparison, such analyses also revealed enhanced and distant bands in regions corresponding to the molecular weight of IDH2 (≈47 kDa) molecules, indicating the upregulation of IDH2 expression in kidneys 3 days after receiving hydrodynamic delivery and 14 days after IPC compared to the levels of IDH2 expression detected in control (sham) kidneys.

### 3.4 Enhanced mitochondrial activity observed in rats treated with IDH2 plasmid DNA and ischemic-preconditioning

**FIGURE 5:**
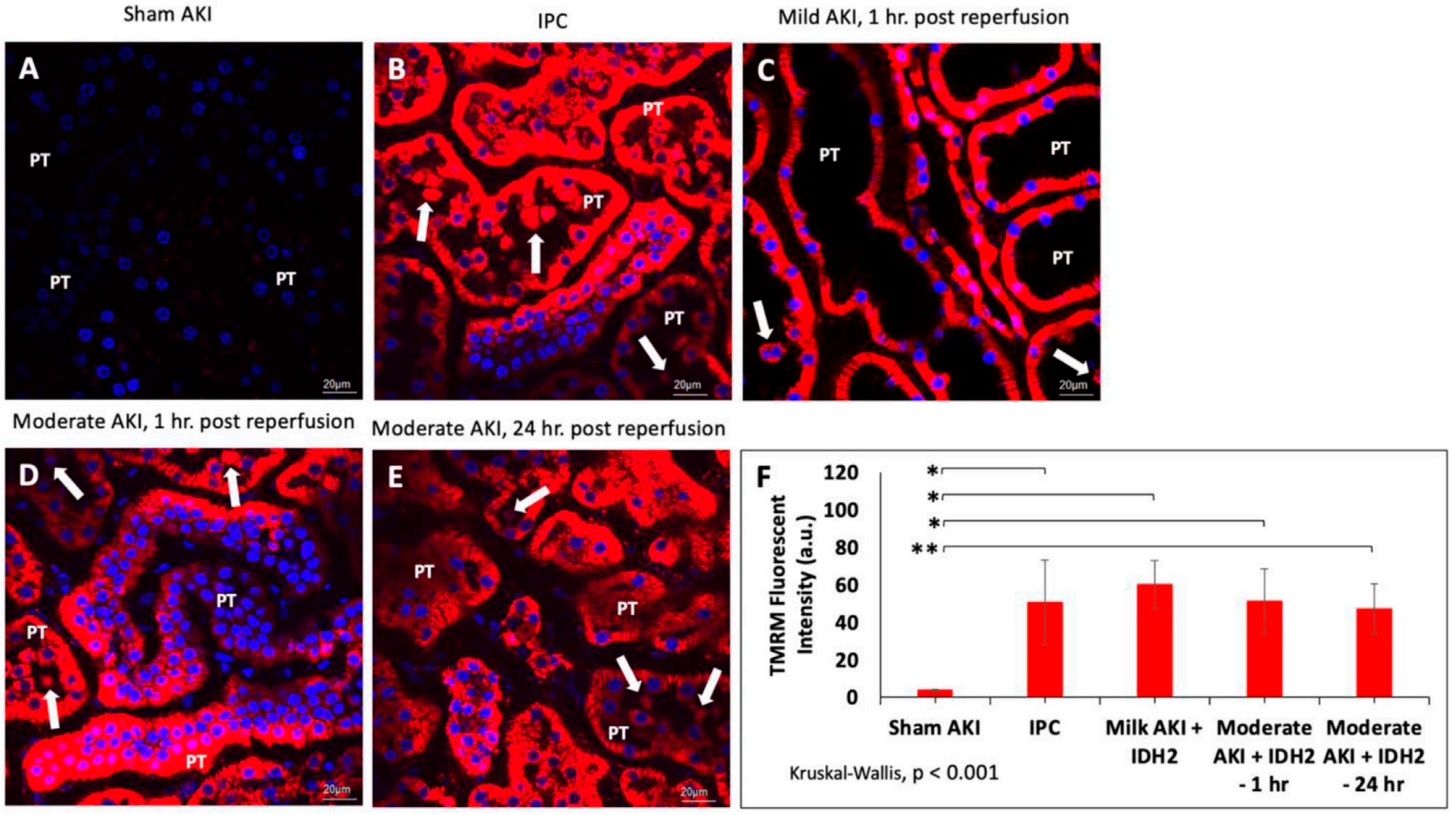
Mitochondrial membrane potential activity was examined in nephron segments of various live rats *via* TMRM-labeled signaling in **(A)** sham animals; **(B)** IPC animals; **(C)** animals with mild AKI that received hydrodynamic injections of IDH2 1 h after inducing IRI for 10–15 min; and **(D)** animals with moderate AKI that received hydrodynamic injections of IDH2 1 h after inducing IRI for 30–35 min; and animals with moderate AKI that received hydrodynamic injections of IDH2 24 h after inducing IRI for 30–35 min. **(E)** The mean TMRM -based fluorescent intensity from each experimental condition (*n* = 10 tubules per rat). The arrows pinpoint fluorescent casts/cellular debris within the tubular lumen. PT, proximal tubule. **(F)** Mean TMRM fluorescent intensities that were recorded from the various experimental groups. Scale bar, 20 μm, * represents *p* < 0.001, and ** represents *p* = 0.001.

In order to gain physiological insight into the effects of IDH2 upregulation in these injury models, mitochondrial activity was investigated in various groups of live rats 3 days after hydrodynamic gene delivery of IDH2 and 14 days after IPC. Intravital measurements of the mean fluorescent intensities were obtained approximately 20 min after the jugular infusion of the mitochondrial membrane potential-dependent dye, TMRM. These investigations revealed substantial elevations in TMRM-based fluorescence signals obtained from kidneys that received IDH2 gene transfer (5C through 5E) and IPC (Figure 5B), compared to the sham group (Figure 5A). Specifically, enhancements in mitochondrial activity exceeded factors of 13 (*p* < 0.001 at T_1hr_) and 11 (*p* < 0.001 at T_24hr_) when IDH2 hydrodynamic-based treatments were applied to 1h and 24 h after inducing moderate AKI.

Such results illustrate a potential increase in mitochondrial activity related to the upregulation of the mitochondrial enzymes facilitated by hydrodynamic-derived gene delivery and ischemic pre-conditioning, which are consistent with the previous studies (Kolb et al., 2018). This upregulation in mitochondrial enzyme activity was confirmed using Western analysis (Figure 4J). Moreover, the combination of Hoechst 33342-based and TMRM-based staining was used to further examine alterations to the innate renal ultrastructure. The probes highlighted cellular sloughing from the tubular epithelium, cast formation within the tubular lumen, and collapsed tubules. Interestingly, such enhancements to the mitochondrial proteome occurred despite substantial deformation to the renal microarchitecture. As anticipated, lower degrees of injury were observed in the animals subjected to sham and mild injuries.

Furthermore, non-parametric statistical analyses using the Kruskal-Wallis (*p* < 0.001) revealed significant differences among these TMRM-based fluorescent intensities. At the same time, Dunn’s *ad hoc* test only indicated significant differences between the means of sham injured rats and rats with mild injuries followed by hydrodynamic gene delivery 1 h after reperfusion (Mild AKI + IDH2—1 h), *p* < 0.001, rats with moderate injuries followed by hydrodynamic gene delivery 1 h after reperfusion (Moderate AKI + IDH2—1 h), *p* < 0.001, and rats with moderate injuries followed by hydrodynamic gene delivery 24 h after reperfusion (Moderate AKI + IDH2—1 h), *p* = 0.001 (Figure 5F).

### 3.5 SCr, BUN, and urine output indicate that hydrodynamic injections of IDH2 plasmids boost recovery and halt the functional progression of AKI despite increased levels of structural damage

**FIGURE 6:**
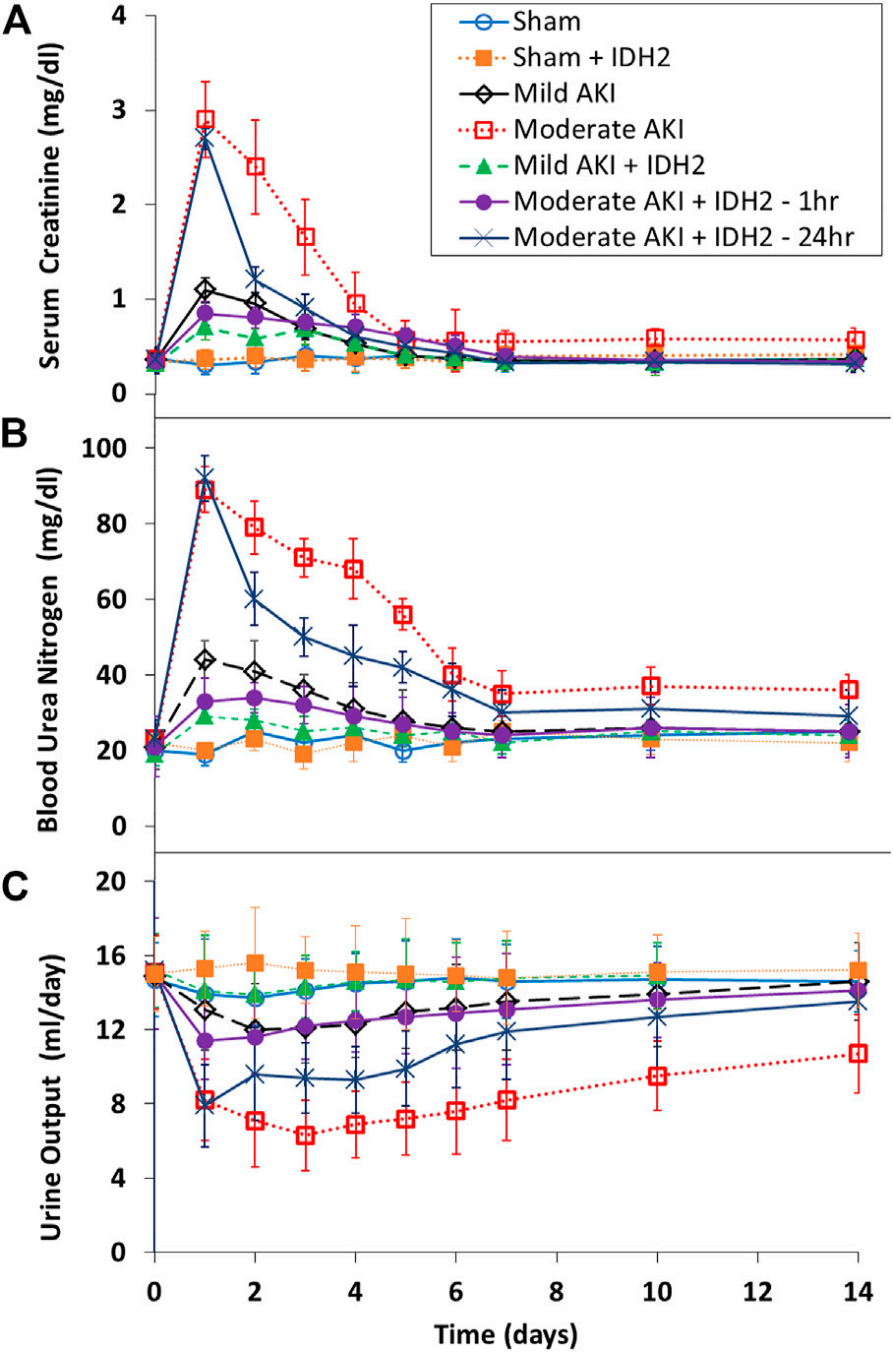
Blood biochemical and urine volumetric analyses. A comparative view of the restorative effects of upregulating IDH2 renal expression *via* hydrodynamic injections on **(A)** serum creatinine, **(B)** blood urea nitrogen, and **(C)** urine across the 14-day measurement period compared to characteristic injury responses. n = 3 for all groups.

Biochemical (Figures 6A, B) and volumetric (Figure 6C) analyses demonstrated the restorative effect that upregulated IDH2 expression provided since, on average, SCr levels (≈60%, *p* < 0.05 at T_1hr_; ≈50%, *p* < 0.05 at T_24hr_) and blood urea nitrogen (≈50%, *p* < 0.05 at T_1hr_; ≈35%, *p* < 0.05 at T_24hr_) were decreased. Furthermore, SCr and BUN measurements show how hydrodynamic IDH2 plasmid delivery 1 and 24 h after inducing moderate IRI in live rats significantly blunted the normal elevated injury response. Strikingly, hydrodynamic plasmids injections administered at the 24-h mark resulted in faster returns to normal baseline levels. This return to baseline occurred as early as 3 days after the initial insult, compared to 7 days in the groups that did not receive hydrodynamic injections of the key mitochondrial enzyme, IDH2. Additionally, providing these injections at the 1-h mark after inducing moderate AKI lowered the variations in these biomarkers significantly and halted the injury’s effect, and creatine and BUN values stayed within the normal range. The data presented an analogous effect on urine output as pairwise comparisons revealed an average increased urine output (≈40%, *p* < 0.05 at T_1hr_; ≈26%, *p* < 0.05 at T_24hr_). The volumetric data appeared to support the urinalysis directly.

**FIGURE 7:**
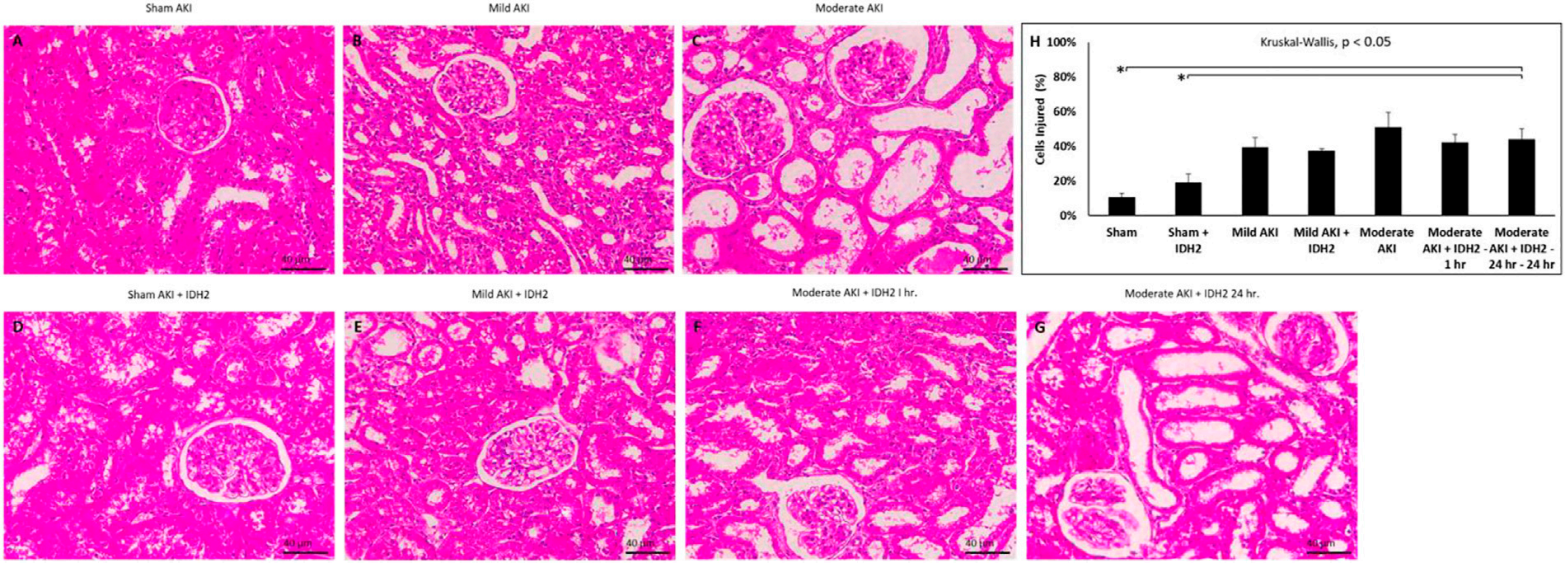
Histological assessments. X60 brightfield images of cortical sections from **(A)** sham AKI, **(B)** mild AKI, **(C)** moderate AKI, **(D)** Sham AKI + IDH2, **(E)** mild AKI + IDH2 (HD 1-h post-reperfusion), **(F)** moderate AKI + IDH2 (HD 1-h post-reperfusion) **(G)** moderate AKI + IDH2 (HD 24-h post-reperfusion). **(H)** Comparison of the injury levels observed in tubular cells from all the above groups. *n* = 3 for all groups. Scale bar, 40 μm *represents significant differences between groups at a level of *p* < 0.001.

Conversely, histological assessments provided further insight into the damage that resulted from these experimental conditions (Figure 7; Table 1). Mild degrees of morphological injury were observed in the Sham AKI (Figure 7A) and mild AKI (Figure 7B) groups. Interestingly, comparable injuries were observed with each corresponding case that accompanied hydrodynamic delivery of IDH2 plasmid vectors, i.e., there were no significant differences between the levels of injury observed between the AKI and AKI + IDH2 groups (*p* ≮ 0.05), and the mild AKI and mild AKI + IDH2 groups (*p* ≮ 0.05). Whereas more substantial levels of structural damage were detected in the animals subjected to moderate forms of injury (Figure 7C). Again, analogous injury levels were also detected between the moderate AKI group and the moderate AKI group that received hydrodynamic IDH2 plasmid injections 1 h (*p* ≮ 0.05) and 24 h (*p* ≮ 0.05) after inducing IRI.

**TABLE 1:** Histological assessments for each experimental condition.

Experimental condition	Average total cell counts	Average number of damaged cells	% Damaged cells
Sham	433	42	9.76
Sham + IDH2	450	62	13.78
Mild AKI	860	328	38.28
Mild AKI + IDH2	450	148	33.22
Moderate AKI	466	265	57.99
Moderate AKI + IDH2–1 h	463	194	42.26
Moderate AKI + IDH2–24 h–24 h	517	156	30.45

Previous characterizations of the injection process performed with vascular cross-clamping have outlined that the hydrodynamic forces and brief ischemia-reperfusion injury generated from this 3-min process are associated with a minor level of injury, far lower than that what is derived from the mild form of AKI, which was induced with 10–15 min of IRI (Corridon et al., 2013). Also, the concentration of the plasmid vectors used in this study is not known to induce appreciable pathogenicity and toxicity. As a result, comparisons made among these groups of animals could be used to demonstrate that the injection process or the transgene expression did not adversely affect morphology. Notably, the degrees of structural damage observed in the sham, mild, and moderate experimental conditions (with and without IDH2 treatments) are consistent with these forms of injury. Thus, it can be inferred, in combination with the SCr and BUN analyses, that no further substantial degree of injury could have resulted from the injection process.

Yet, extensive disruptions to proximal tubular brush border integrity and distal tubular and glomerular deformation produced significantly elevated average histology injury scores (≈40%, *p* < 0.05 at T_1hr_; ≈40%, *p* < 0.05 at T_24hr_). These results coincide with the fact that renal IRI is a dynamic process characterized by endothelial and epithelial cellular injury, inflammation, and hemodynamic alterations (Bonventre and Yang, 2011). Within proximal nephron segments, several essential and high energy demand ATP-dependent transport processes are eliminated by this form of injury (Vormann et al., 2022). Typically, the proximal tubule is the main site of injury in renal IRI, and the injuries observed herein coincide with reductions in oxygen associated with diminished renal blood flow. While this segment contains a high density of mitochondria, it lacks anaerobic ATP-generating capacity and is thus quite vulnerable to such oxygen supply disruptions (Hall and Molitoris, 2014).

Even though IRI is widely associated with tubular damage, this form of injury also induces significant podocyte effacements and disruptions of slit diaphragms (Wagner et al., 2008), which can lead to atypical filtrative capacities observed in these intravital studies. On the other hand, distal tubules have far greater glycolytic capacities than their proximal counterparts, which supports their ability better to maintain the Δψ_m_ and mitochondrial structure during ischemia (Hall et al., 2009). Likewise, prerenal flow reductions can also reduce GFR by limiting glomerular perfusion and increasing tubular obstruction due to necrotic and sloughed tubular epithelial cells (Burek et al., 2020), as observed in the intravital micrographs (Figure 3 through Figure 5).

## 4 Discussion

AKI is a severe medical condition that results in approximately 1.7 million deaths worldwide each year (Vormann et al., 2022). Treatments that can rapidly restore renal function are attractive options to help reduce the global burden of kidney diseases. Current AKI treatment regimens are mainly supportive and cannot halt the illness’s progression into chronic and end-stage conditions. Thus, there is a need for alternative treatments, like gene therapy (Pantic et al., 2022a; Wang et al., 2023; Pantic et al., 2023a; Khan et al., 2023). This form of therapy provides a means to treat and prevent the root cause of a given condition. Over the years, several methods have been proposed to elicit efficient gene transfer in various settings (Corridon, 2022b; Corridon et al., 2022; Davidovic et al., 2022; Wang et al., 2022a; Wang et al., 2022b; Corridon, 2023; Pantic et al., 2023b; Shakeel and Corridon, 2023). However, progress in the development of direct gene transfer methods in the kidney has been limited compared with other organs. This issue is partly due to the need to understand better the biological factors needed to promote efficient vector entry into the cell and the complex anatomical structure of the kidney (Davis and Park, 2019).

Recently, plasmid-based hydrodynamic gene delivery has shown promise in overcoming this issue (Corridon, 2023). Therefore, this study aimed to demonstrate the potential of hydrodynamic-based gene delivery to halt progressive or persistent renal function impairment following episodes of ischemia-reperfusion injuries. The approach presented in this manuscript is based on the transfer of an essential enzyme *via* a plasmid vector at the inception of IRI-based injuries to boost renal recovery. In this first instance, it was essential to identify whether this rapid injection technique, augmented with vascular cross-clamping, could provide widespread and targeted renal vector delivery.

In order to achieve this goal, various attempts were made to examine the possibility of extending previous successful applications using this delivery technique to upregulate the level of mitochondrial enzymatic activity. From a mechanistic perspective, hydrodynamic injections of 0.5 mL of tolonium chloride solutions revealed robust distributions of this pigment across the injected kidney and no visible traces within the right contralateral kidney. Additionally, the localization of the large molecular weight dextran molecules within the damaged tubular epithelium, illustrated the potential to facilitate the cellular internalization of exogenous plasmids of comparable size using this technique (Corridon et al., 2013; Nishi et al., 2017). These macroscopic and microscopic evaluations provided sufficient evidence that the injection process could facilitate widespread and targeted delivery of the vector to the kidney.

The next phase of the study focused on the actual delivery of plasmid vectors and their resulting expression rates, using EGFP-actin reporter plasmids. IVM also allowed the *in vivo* visualization of the reporter transgene expression, and it was reassuring to detect significant and comparable rates of fluorescent protein expression in the injury models, especially for the moderate forms of AKI. From the estimated transfection rates in the injured rats, it is conceivable that ischemia-reperfusion injury, which is known to promote vasculature and cellular permeability, may have afforded an increased passage of exogenous material across denatured membranes. This phenomenon may have also directly enhanced the internalization of plasmids/exogenous probes in the possible absence or reduction of innate endocytic capacities and supports a similar rationale for the transgene expression observed in rats with injured glomeruli using viral vectors (Kelley and Sukhatme, 1999).

Earlier on, hydrodynamic gene delivery was initially conducted on mice. However, there is a clear advantage in performing renal-targeted hydrodynamic gene delivery in rats compared to mice due to the relative ease in executing intricate intravascular injections in rats and inducing hemostasis post-injection without inducing further bouts of ischemic injury and damage to the smaller and more delicate mouse renal vein. Moreover, a growing body of evidence suggests that gender differences exist in cardiovascular responses to significant alterations to normal circulatory conditions, including vascular occlusive cases and hypertension (Lü et al., 2005; Gao et al., 2019). While the underlying mechanism(s) related to these gender differences remain to be elucidated, the author believed that it was essential to conduct this study using male rats to provide a proper comparison for previous work that led to the design of this investigation.

Also, it is well known that AKI is strongly associated with damage to the proximal tubule and that the dysfunction or death of proximal tubule cells is often the main consequence of AKI (Corridon et al., 2021). Cells within this epithelium often undergo programmed cell death or necrosis depending on the severity of the injury, while in some very mild AKI cases, such cellular dysfunction is barely noticeable and reversible without intervention (Corridon et al., 2013). This investigation captured the intrinsic nature of this form of injury, as the real-time effects of cellular damage led to the effacement and loss of brush border in the proximal tubule, detachment of tubular epithelial cells from associated basement membranes, and cellular sloughing, which resulted in the formation of fluorescent casts with tubular lumens (Basile et al., 2012; Corridon et al., 2021). Besides, prerenal injury generates immediate and marked rises in the nicotinamide adenine dinucleotide (NADH) signal in tubular cells, which then decrease again upon restoration of renal blood flow and imply rapid transitions that occur in tubular cellular mitochondria activity stemming from IRI. This conversion can lead to decreased respiratory chain activity and a shift of the NADH pool into a reduced state (Hall and Molitoris, 2014), thereby adversely reducing the energy carrier capacity within the nephron.

At this stage, examining the therapeutic potential of upregulating IDH2 activity in these injury models was necessary. The impacts of such genetic alterations on renal function were evaluated using various biochemical, microscopic, and volumetric analyses. Western blot analyses confirmed enhanced expression of this crucial mitochondrial enzyme after hydrodynamic gene transfer. Likewise, before any injury, serum creatinine (SCr), blood urea nitrogen (BUN), and urine output aligned with respective typical ranges of 0.4–0.8 mg/dl^90^, 15–22 mg/dL (Thammitiyagodage et al., 2020), and 10–15 mL/day (Kurien et al., 2004). Conversely, with AKI, sudden and significant spikes in SCr and BUN were observed and known to usually occur within 24 h of the inception of the injury, whereas these levels gradually returned to baseline (Zhou et al., 2006; O’Kane et al., 2018).

Remarkably, the major finding in this study is that the hydrodynamic-based mitochondrial IDH2 gene delivery led to recovery and halt of the progression of AKI by decreases in serum creatinine and blood urea nitrogen levels, and increases in urine output and mitochondrial membrane potential. In contrast, the introduction of IRI produced representative and inverse effects on urine production (Wei et al., 2019) and mitochondrial activity (Hato et al., 2018). Although the metabolic cage is commonly used for such studies, it has been recognized as constituting a unique stressor that could affect behavior and the measurements presented (Kalliokoski et al., 2013; Whittaker et al., 2016). This effect is a possible limitation of the study that can be examined in future research by comparing the results obtained in this study with conventional housed rats. Recognizing this issue and after receiving approval for such studies, the animals were checked daily by the researcher and facility staff for signs of stress, and if any adverse conditions were identified in a given animal, it would have been excluded from the study.

Recalling that mitochondrial impairment resulting from IRI represents an early step in the pathogenesis of renal injury, harnessing the therapeutic effects observed in IPC may be the key to recovery. Specifically, from a molecular perspective, mitochondrial isocitrate dehydrogenases catalyze the oxidative decarboxylation of isocitrate. These enzymes are integral to cellular mechanisms that have evolved to shield cells from oxidative damage (Zhang et al., 2013) and the unique acceleration of renal senescence due to IDH2 depletion (Lee et al., 2017). Such effects are partly mediated by preserving mitochondria function in response to IRI by preserving cellular ATP levels, reducing reactive oxygen species (ROS) production, and inhibiting cell death pathways (Jassem et al., 2002; Kolb et al., 2018). We had already shown that gene delivery of IDH2 before injury attenuated surges in serum creatinine and amplified the mitochondria membrane potential, maximal respiratory capacity, and intracellular ATP levels (Kolb et al., 2018). Furthermore, this delivery process has also been shown to improve microvascular perfusion by eliminating rouleaux to reduce peritubular capillary erythrocyte congestion and the accumulation of renal leukocytes (Collett et al., 2017). Therefore, it is reasonable to attribute comparable mechanistic responses generated by cellular pathways that supported renal recovery amidst substantial morphological damage.

## 5 Conclusion

Traditionally, the development of renal gene therapy has lagged other organ-directed gene therapies due to low renal gene transfer efficiencies and difficulty targeting specific kidney cell types. Nevertheless, hydrodynamic delivery appears to overcome this problem in the setting of mild and moderate acute disorders. This possibility may benefit the outcome of AKIs, characterized by sudden losses of renal function, subsequent rises in creatinine and BUN, and reductions in urine output, as current treatments are mainly supportive. It is possible to arrive at this conclusion from the data presented in this report, as this relatively simple technique could be used to generate efficient transgene expression in live renal segments, namely tubular epithelial cells, which are major sites of damage in AKI (Molitoris, 2004; Chevalier, 2016; Corridon et al., 2021). Interestingly, substantial levels of transgene expression were observed in mammalian kidneys with AKI during both the initial phase of injury and at the maximal point of damage, albeit in a limited sample size.

Based on this, it is necessary to consider the state of the cells expressing the transgenes. There is a high probability that injured, and functional cell populations would express transgenes in some undefined ratio. Naturally, it would be of interest to shift this ratio to benefit injured cells. Nevertheless, due to the nature of AKI and proximal tubule function, it would be of interest to target functional cells in an attempt to protect them from irreversible damage and foster their proliferation to compensate for terminally injured cells (Kume et al., 2012). Hence, the results presented herein may be used to further investigate the delivery of clinically relevant transgenes in an attempt to provide further therapeutic, yet transient genetic modifications in live mammalian kidneys. Such approaches can improve the current understanding of and ways to combat AKI by boosting recovery and halting progression at its inception. Although gene therapy for kidney disease remains a major challenge, new and evolving technologies may actualize treatment for AKI (Peek and Wilson, 2022). Continued and complimentary research to identify new key structural and functional genetic targets, and better examine existing ones while improving gene delivery, will further enhance the utility of genetic medicine as we aim to envision its promise.

Finally, the versatility and ease of use of the CRISPR gene editing system also suggest its potential for renal gene therapy. CRISPR has been shown to work in mammalian (including human) cells, underlining the possibility of using this technique for clinically driven gene editing and gene targeting applications (Cruz and Freedman, 2018; Aulicino et al., 2022). As with previous rodent studies, systemic delivery (*via* the tail vein) of CRISPR editing tools provided evidence of genetic variations limited to the liver (Liang et al., 2022). Thus, as with other vectors, significant challenges remain, including how to effectively deliver CRISPR to kidneys and control gene editing events within the genome (Cruz and Freedman, 2018). Future studies could be devised for the combination of retrograde renal hydrodynamic fluid delivery and CRSPR to devise preclinical models that forge its safe and efficacious translation into therapies.

## Data Availability

The original contributions presented in the study are included in the article/supplementary materials, further inquiries can be directed to the corresponding author.
